# Inositol 1,4,5‐trisphosphate receptor determines intracellular Ca^2+^ concentration in *Trypanosoma cruzi* throughout its life cycle

**DOI:** 10.1002/2211-5463.12126

**Published:** 2016-10-14

**Authors:** Muneaki Hashimoto, Motomichi Doi, Nagomi Kurebayashi, Koji Furukawa, Hiroko Hirawake‐Mogi, Yoshihiro Ohmiya, Takashi Sakurai, Toshihiro Mita, Katsuhiko Mikoshiba, Takeshi Nara

**Affiliations:** ^1^ Department of Molecular and Cellular ParasitologyJuntendo University School of MedicineTokyoJapan; ^2^ Health Research InstituteAISTTakamatsuKagawaJapan; ^3^ Biomedical Research InstituteAISTTsukubaIbarakiJapan; ^4^ Department of PharmacologyJuntendo University School of MedicineTokyoJapan; ^5^ Laboratory for Developmental NeurobiologyRIKEN Brain Science InstituteSaitamaJapan

**Keywords:** intracellular Ca^2+^ concentration, IP_3_ receptor, life cycle, live cell Imaging, *Trypanosoma cruzi*

## Abstract

Regulation of intracellular Ca^2+^ concentration ([Ca^2+^]_i_) is vital for eukaryotic organisms. Recently, we identified a Ca^2+^ channel (TcIP_3_R) associated with intracellular Ca^2+^ stores in *Trypanosoma cruzi*, the parasitic protist that causes Chagas disease. In this study, we measured [Ca^2+^]_i_ during the parasite life cycle and determined whether TcIP_3_R is involved in the observed variations. Parasites expressing R‐GECO1, a red fluorescent, genetically encoded Ca^2+^ indicator for optical imaging that fluoresces when bound to Ca^2+^, were produced. Using these R‐GECO1‐expressing parasites to measure [Ca^2+^]_i_, we found that the [Ca^2+^]_i_ in epimastigotes was significantly higher than that in trypomastigotes and lower than that in amastigotes, and we observed a positive correlation between *TcIP*_*3*_ *R* mRNA expression and [Ca^2+^]_i_ during the parasite life cycle both *in vitro* and *in vivo*. We also generated R‐GECO1‐expressing parasites with TcIP_3_R expression levels that were approximately 65% of wild‐type (wt) levels (SKO parasites), and [Ca^2+^]_i_ in the wt and SKO parasites was compared. The [Ca^2+^]_i_ in SKO parasites was reduced to approximately 50–65% of that in wt parasites. These results show that TcIP_3_R is the determinant of [Ca^2+^]_i_ in *T. cruzi*. Since Ca^2+^ signaling is vital for these parasites, TcIP_3_R is a promising drug target for Chagas disease.

**Abbreviations**
[Ca^2+^]_i_: intracellular Ca^2+^ concentration
IP_3_Rs: inositol 1,4,5‐trisphosphate receptors
R‐GECO1: red fluorescent, genetically encoded Ca^2+^ indicator for optical imaging
SKO: single‐knockout
TcIP_3_R: IP_3_R homolog in *T. cruzi*

Calcium ion (Ca^2+^) is the most important and versatile intracellular messenger in eukaryotes 1. Ca^2+^ signaling regulates various biological process, including secretion, fertilization, cell growth, and cell death 2; thus, the regulation of intracellular Ca^2+^ concentration ([Ca^2+^]_i_) is vital. In mammals, [Ca^2+^]_i_ is regulated by several factors, including Ca^2+^ influx into the cytosol through voltage‐gated Ca^2+^ channels (VGCCs), receptor‐operated Ca^2+^ channels (ROCs), and store‐opened Ca^2+^ channels (SOCs); buffering of Ca^2+^ with plasma membrane and cytosolic proteins; accumulation of Ca^2+^ in intracellular Ca^2+^ stores through the sarcoplasmic/endoplasmic reticulum Ca^2+^‐ATPase (SERCA); and efflux of Ca^2+^ from stores through Ca^2+^ channels, such as inositol 1,4,5‐trisphosphate receptors (IP_3_Rs), and ryanodine receptors (RyRs) 3.

*Trypanosoma cruzi* is the parasitic protist that causes Chagas disease in Latin America. At present, only two drugs are available for Chagas disease (benznidazole and nifurtimox), and these often induce severe side effects and are effective for only the acute phase of the disease. Since no practical drug or vaccine for Chagas disease is available, new treatments are greatly needed 4. The life cycle of the parasite comprises two phases, the insect and mammalian phases 5. In the insect vector (the reduviid bug), the epimastigote replicates and transforms into a metacyclic trypomastigote (metacyclogenesis). A nonproliferating metacyclic trypomastigote invades a mammalian host, and is then transformed into an amastigote inside a wide variety of nucleated cells. The intracellular amastigote multiplies by binary fission, and is then transformed back into a trypomastigote, which is released into the circulation after host cell disruption.

[Ca^2+^]_i_ regulation is vital for *T. cruzi*, and the molecular mechanisms of [Ca^2+^]_i_ regulation in the parasite are thought to be quite different from those in mammalian cells 6. In fact, no homologs of the typical Ca^2+^ transporters—ROCs, SOCs, or Na^+^/Ca^2+^ exchangers, have been detected in Trypanosomes. The results of a proteome analysis of *T. brucei* suggested that a putative VGCC is localized to the flagellum 7. Two homologs of plasma membrane Ca^2+^ ATPase (PMCA) have also been reported; one is localized on the plasma membrane, and the other is localized to the acidocalcisome of *T. brucei* 8. In addition, a SERCA has been shown to be localized to the ER of *T brucei* like mammalian cells 9. However, no RyR homologs have been reported. Recently, we identified an IP_3_R homolog in *T. cruzi* (TcIP_3_R), and showed that it is mainly localized to the ER. When the expression level of TcIP_3_R is reduced to less than one‐half of that in wild‐type (wt) cells, the parasite cannot grow 10. Therefore, TcIP_3_R may be a promising drug target 11. We also previously showed that TcIP_3_R regulates parasite growth, transformation, infectivity, and virulence in mammalian hosts, indicating that TcIP_3_R is an important regulator of the parasite life cycle 10, 12. In fact, experiments using classical Ca^2+^ indicators, such as Fura‐2, showed that Ca^2+^ signaling is important for host cell invasion 10, 13, 14 as well as proliferation and transformation 15.

In this paper, we reported the successful preparation of parasites expressing R‐GECO1 (a red fluorescent, genetically encoded Ca^2+^ indicator for optical imaging), which is a green fluorescent protein (GFP) variant that fluoresces only upon binding to Ca^2+^ 16. It has recently been reported that other parasites including *Plasmodium falciparum* and *Toxoplasma gondii* that express genetically encoded Ca^2+^ indicators are useful for investigating Ca^2+^ signaling in the parasite 17, 18. Importantly, our findings revealed that analysis of *T. cruzi* expressing R‐GECO1 revealed that the [Ca^2+^]_i_ in the parasite changes significantly during its life cycle, and that TcIP_3_R is the determinant of [Ca^2+^]_i_ in *T. cruzi*.

## Materials and methods

### Plasmid construction

The *R‐GECO1* gene was amplified by PCR from the pCMV‐R‐GECO1 plasmid vector (Addgene, plasmid 45494) using specific primers (forward: 5′‐CACCATGGTCGACCTTCACGTCGTA‐3′ and reverse: 5′‐CTACTTCGCTGTCATCATTTGTAC‐3′; the CACC sequence required for directional cloning in pENTR/D‐TOPO is underlined) and KOD‐Plus Neo (TOYOBO Co., Ltd, Osaka, Japan). The PCR‐amplified gene was inserted into pENTR/D‐TOPO (Life Technologies, Rockville, MD, USA). The resultant plasmid, pENTR/*R‐GECO1*, was converted to a pTREX vector 19, 20, which contains a neomycin resistance gene as the selection marker, and was modified by the Gateway Vector Conversion System (pTREX(neo^R^)‐GW; Life Technologies) using the Gateway recombination system, to generate pTREX (neo^R^)‐GW/*R‐GECO1*.

The puromycin resistance gene was amplified by PCR using pBApo‐CMV Pur DNA plasmid vector (Clontech Laboratories, Inc., Mountain View, CA, USA) as the template with the specific primers (forward: 5′‐ATGACCGAGTACAAGCCCAC‐3′ and reverse: 5′‐TCAGGCACCGGGCTTGC‐3′). To remove the neomycin resistance gene from pTREX (neo^R^)‐GW/*R‐GECO1*, we used PCR with pTREX (neo^R^)‐GW/*R‐GECO1* as the template and the primers (forward: 5′‐GGGGATCGATCCGGAACAA‐3′ and reverse: 5′‐ATTGGCTGCAGGGTCGCT‐3′). These two PCR fragments were ligated with DNA Ligation Kit Ver. 2.1 (Takara Bio Inc., Shiga, Japan), to generate pTREX (pur^R^)‐GW/*R‐GECO1*.

### Cell culture

Epimastigotes of the *T. cruzi* Tulahuen strain were cultured as previously described 21. The mammalian stages of the parasites were maintained in HeLa cells or 3T3‐Swiss albino cells (Health Science Research Resources Bank, Tokyo, Japan), and trypomastigotes were collected from subcultures of infected 3T3‐Swiss albino cells by centrifugation as previously described 22. Metacyclogenesis was performed as previously described 23. Quantitative real‐time RT‐PCR analysis was performed as previously described 10. 1,2‐Bis(2‐aminophenoxy)ethane‐*N,N,N*′,*N*′‐tetraacetic acid (BAPTA), which is a cell‐impermeant Ca^2+^ chelator and reduces the levels of extracellular Ca^2+^, and 1,2‐Bis(2‐aminophenoxy)ethane‐*N,N,N′,N′*‐tetraacetic Acid, tetraacetoxymethyl ester (BAPTA‐AM), which is a cell‐permeant Ca^2+^ chelator thereby reduces [Ca^2+^]_i_, were purchased from Dojindo Molecular Technologies, Inc. (Kumamoto, Japan), and an IP_3_R inhibitor, 2‐aminoethoxydiphenyl borate (2‐APB), was purchased from Sigma‐Aldrich (St. Louis, MO, USA).

### Expression of R‐GECO1 in *T. cruzi*

A total of 1 × 10^7^ epimastigotes were resuspended in Amaxa Basic^®^ Parasite Nucleofector Kit 2 solution (Lonza, Köln, Germany). The resuspended wt or TcIP_3_R SKO parasites 10 were mixed with 10 μg of pTREX (neo^R^)/*R‐GECO1* or pTREX (pur^R^)/*R‐GECO1*, respectively, and then electroporated with an Amaxa Nucleofector Device (Lonza) using program U‐033. Stable wt transformants expressing R‐GECO1 were selected by incubating the cells for 30 days in LIT medium containing 0.5 mg·mL^−1^ G418, and then clonal derivatives were isolated by limiting dilution. The stable SKO transformants expressing R‐GECO1 were selected by incubating the cells for 30 days in LIT medium containing 0.25 mg·mL^−1^ G418 and 3 μg·mL^−1^ puromycin. Amastigotes or trypomastigotes stably expressing R‐GECO1 were transformed from the epimastigotes expressing R‐GECO1 using the methods described above.

### Detection of [Ca^2+^]_i_ by fluorescence microscopy

Fluorescence images of the parasites were acquired using a fluorescent microscope (Axio Imager M2; Carl Zeiss Co. Ltd., Oberkochen, Germany) or a laser confocal microscope (Nikon A1R, Nikon Co. Ltd., Tokyo, Japan). After acquiring fluorescence images in normal culture medium, the maximal fluorescence signal (*F*_max_) of R‐GECO1 in individual parasites was determined by treating them with high Ca^2+^ solution (10 mm) and ionomycin (0.26 mm; Nacalai Tesque, Kyoto, Japan), which increase the cytosolic Ca^2+^ content of parasites so as to saturate R‐GECO1 with Ca^2+^. To convert fluorescence intensity to Ca^2+^ concentration, the following formula was used:

$${{\left[{\text{Ca}}^{2+}\right]}_{i}}^{.5n}=K_{d}^{n}\times \left(F-F_{\mathrm{MIN}}\right)/\left(F_{\mathrm{MAX}}-F\right)$$

where *K*_d_ is the dissociation constant for R‐GECO1 (482 nm); *n* is the Hill coefficient of R‐GECO1 (2.0); *F* is the fluorescence intensity in each parasite; *F*_MAX_ is the maximal fluorescence intensity of R‐GECO1 in the parasite (see above); and *F*_MIN_ is the minimal intensity of R‐GECO1 calculated using the ratio change value obtained by Zhao *et al*. (= 1/16 of *F*_MAX_).

### Statistical analysis

Statistical analysis was performed with sigma plot ver. 12 software (Systat Software, Inc., San Jose, CA, USA) using one‐way ANOVA or Student's *t*‐test.

## Results and Discussion

### The [Ca^2+^]_i_ changes significantly during the life cycle of *T. cruzi*

**Figure 1:**
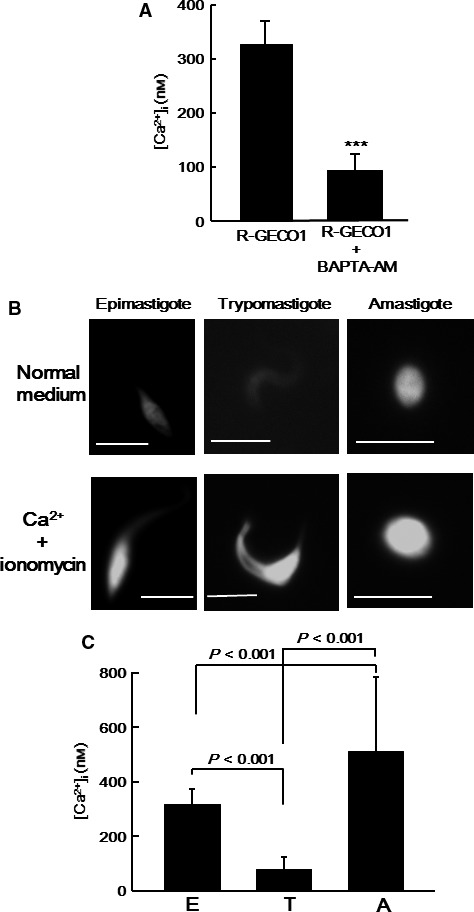
Changes in *T. cruzi* [Ca^2+^]_i_ during its life cycle. (A) The fluorescence intensity of epimastigotes expressing R‐GECO1 was measured after treatment with 100 μm BAPTA‐AM for 3 min. The [Ca^2+^]_i_ were calculated from the fluorescence intensity and compared to that in untreated epimastigotes. The fluorescence intensity of 20 parasites for each condition was measured. Statistical analysis between the two groups was performed using Student's *t*‐test. ***P < 0.001. (B) Typical images of a clonal derivative of *T. cruzi* expressing R‐GECO1 in normal culture medium (top) and in high Ca^2+^ medium with ionomycin (bottom), including an epimastigote, trypomastigote, and amastigote, are shown. *Bar*, 5 μm. (C) The [Ca^2+^]_i_ in epimastigotes (E), trypomastigotes (T), and amastigotes (A), as measured by fluorescence intensity, was compared. The fluorescence intensity of 20 parasites was measured for each stage. Statistical analysis between the groups was performed using one‐way ANOVA and Tukey's Test.

To investigate the changes in the [Ca^2+^]_i_ in *T. cruzi* during its life cycle, parasites expressing R‐GECO1 were prepared. The *R‐GECO1* gene was cloned in the *T. cruzi* pTREX expression vector 19, in which *R‐GECO1* is expressed under the ribosomal promoter; therefore, R‐GECO1 was constitutively expressed throughout the parasite life cycle. We at first investigated whether the fluorescence signal in the parasites expressing R‐GECO1 was reduced after treatment with a cell‐permeant Ca^2+^ chelator BAPTA‐AM. After replacement of the parasite medium with PBS, BAPTA‐AM (final concentration 100 μm) was added to the PBS to reduce Ca^2+^, and [Ca^2+^]_i_ was measured after 3 min (Fig. 1A). Treatment of the parasites with BAPTA‐AM significantly reduced the parasite [Ca^2+^]_i_, indicating that R‐GECO1 works as a Ca^2+^ indicator in the parasites. We also found that the parasites were killed by the treatment.

To exclude variations in signal intensity among the parasite clones, a clonal derivative was isolated and used for the experiments. Figure 1B shows the R‐GECO1 signal in the epimastigote, trypomastigote, and amastigote. Although the signal was detected throughout the cytoplasm of the parasites at all stages, the signal intensity was quite different among the different life cycle stages, and the maximal fluorescence intensities of R‐GECO1 obtained in the presence of 260 μm ionomycin and 10 mm Ca^2+^ were similar. The [Ca^2+^]_i_ was calculated from the R‐GECO1 signal intensity and compared among the three stages, as described in the Methods section (Fig. 1C). The [Ca^2+^]_i_ was clearly higher in amastigotes (579 ± 204 nm) and epimastigotes (327 ± 44 nm) than in trypomastigotes (85 ± 39 nm). These results indicated that the [Ca^2+^]_i_ changes significantly during the progression of the parasite life cycle. Ca^2+^ oscillation was not detected at any stage.

### Live cell imaging revealed changes in the [Ca^2+^]_i_ of *T. cruzi* parasitizing host cells

**Figure 2:**
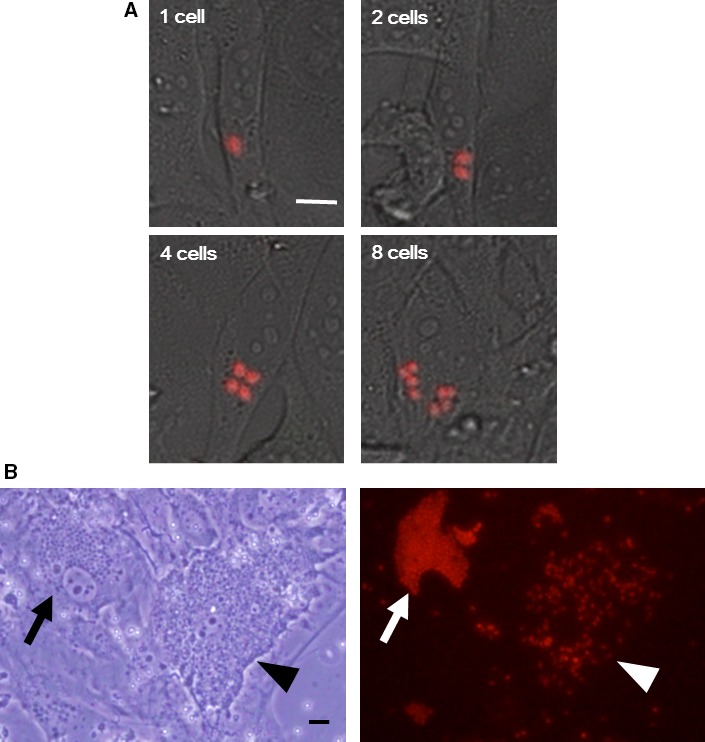
Changes in *T. cruzi* [Ca^2+^]_i_ within host cells. (A) 3T3‐Swiss albino cells were infected with R‐GECO1‐expressing trypomastigotes (red), and imaged 84 h after infection. The movement of an amastigote was recorded using real‐time confocal microscopy with 40× dry objective lens (Nikon AIR). The time interval of the serial images was 15 min. The amastigotes (one cell) that divided once (two cells), twice (four cells), and three times (eight cells) are shown. *Bar*, 5 μm. (B) A bright‐field image of cells that are heavily infected with R‐GECO1‐expressing amastigotes (arrow) and trypomastigotes (arrowhead) is shown (left). A representative microscopic image was obtained with an inverted microscope (IX72; Olympus, Tokyo, Japan). A video of the same field is available as Movie S1. Note that the trypomastigotes in the host cell move intensely. A fluorescent image of the same field is also shown (right). *Bar*, 10 μm.

We further investigated the changes in the [Ca^2+^]_i_ of parasites during intracellular growth. 3T3‐Swiss cells were infected with R‐GECO1‐expressing trypomastigotes, and then the growth of one parasite was monitored under a fluorescent microscope (Fig. 2A). We successfully monitored them until the parasite divided three times within a host cell. The results showed that the [Ca^2+^]_i_ in amastigotes did not change significantly after division.

Amastigotes divide several times within host cells, transform into trypomastigotes, and then lyse the host cells. We investigated whether the [Ca^2+^]_i_ in trypomastigotes parasitizing host cells was decreased similar to that observed in tissue culture‐derived trypomastigotes (Fig. 2B, Movie S1). The results showed that the [Ca^2+^]_i_ in trypomastigotes within host cells (Fig. 2B, arrowhead in the right panel) was significantly lower than that in amastigotes (Fig. 2B, arrow in the right panel).

These results indicate that amastigotes are able to maintain [Ca^2+^]_i_ even in environments where the Ca^2+^ concentration is very low, such as the cytosol of host cells, and that the [Ca^2+^]_i_ in the trypomastigote when inside host cells is significantly lower than that in the amastigote [Ca^2+^]_i_.

### TcIP_3_R is the determinant of [Ca^2+^]_i_ in *T. cruzi*

**Figure 3:**
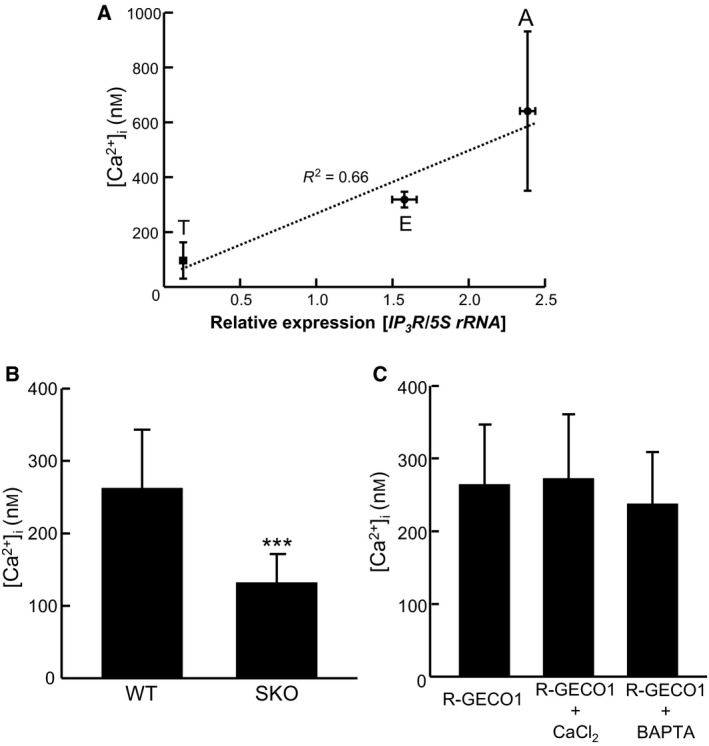
Effect of Ca^2+^ chelators or reduced TcIP_3_R expression on *T. cruzi* [Ca^2+^]_i_. (A) Correlation between *TcIP*_*3*_ *R* mRNA expression level and [Ca^2+^]_i_ in *T. cruzi* throughout the parasite life cycle. [Ca^2+^]_i_ shows a linear relationship with *TcIP*_*3*_ *R* mRNA expression (*R* ^2^ = 0.66). To measure [Ca^2+^]_i_, the R‐GECO1 fluorescence intensity of 20 parasites was measured. To measure *TcIP*_*3*_ *R* mRNA expression, quantitative real‐time RT‐PCR analysis of relative transcript levels was performed, and the data shown are the mean ± SD of three independent experiments. (B) [Ca^2+^]_i_ was compared between R‐GECO1‐expressing wt and SKO epimastigotes. The fluorescence intensity of 20 parasites was measured. Statistical analysis between the groups was performed using Student's *t*‐test. ***P < 0.001. (C) R‐GECO1‐expressing epimastigotes were treated with 10 mm CaCl_2_ or 10 mm BAPTA for 2 h; fluorescence was measured, and [Ca^2+^]_i_ was calculated and compared to that in untreated parasites. The fluorescence intensity of 20 parasites was measured.

Previously, we reported that *TcIP*_*3*_ *R* mRNA expression varied significantly among the parasite life cycle stages 10. Here, we investigated a possible correlation between *TcIP*_*3*_ *R* mRNA expression and the [Ca^2+^]_i_ of the parasite (Fig. 3A). There was a positive correlation between the parasite [Ca^2+^]_i_ at each life stage and the *TcIP*_*3*_ *R* mRNA expression level (*R* ^2^ = 0.66). These results suggest that TcIP_3_R is important for the regulation of [Ca^2+^]_i_ in *T. cruzi*.

To investigate whether TcIP_3_R is involved in the regulation of [Ca^2+^]_i_ in *T. cruzi*, the level of *TcIP*_*3*_ *R* was reduced in parasites expressing R‐GECO1, and the [Ca^2+^]_i_ in wt and mutant parasites was measured (Fig. 3B). We previously found three *TcIP*_*3*_ *R* genes in the genome of the *T. cruzi* Tulahuen strain, and we prepared single‐knockout (SKO) parasites, in which one of the *TcIP*_*3*_ *R* genes was disrupted by homologous recombination 10. We observed the specific disruption of only one *TcIP*_*3*_ *R* gene by Southern blot analysis and an approximately 35% reduction in TcIP_3_R expression levels in the SKO parasites, and these parasites show various phenotypes, such as inhibition of epimastigote growth 10. Since the knockout cassette used to prepare the SKO parasites contained a neomycin resistance gene, the transformants were selected with G418. Then, the *R‐GECO1* gene was cloned into an expression plasmid vector for *T. cruzi* containing a puromycin resistance gene (pTREX(pur^R^)), and then this plasmid was transfected into the SKO parasites. SKO parasites expressing R‐GECO1 were selected in culture medium containing G418 and puromycin. For the control, wt Tulahuen strain parasites were transfected with pTREX(pur^R^)/*R‐GECO1* and selected in culture medium containing puromycin. Since the expression level of R‐GECO1 among the parasite clones might vary, the fluorescence intensity in the parasites was randomly measured without cloning. The fluorescence intensity in the SKO parasites was significantly lower than that in the wt parasites. Importantly, the TcIP_3_R expression level in the SKO parasites was reduced to approximately 65% of wt levels 10, and the R‐GECO1 signal in SKO parasites was reduced to approximately 50% of wt levels.

Next, we investigated whether Ca^2+^ influx from the extracellular fluid or efflux to the extracellular fluid is important for maintenance of [Ca^2+^]_i_ (Fig. 3C). Excessive amounts of CaCl_2_ or BAPTA, a noncell‐permeable Ca^2+^ chelator, was added to the cultivation medium of epimastigotes expressing R‐GECO1, and the [Ca^2+^]_i_ in treated parasites was compared to that in untreated parasites after 2 h. No increase was detected in parasites treated with CaCl_2_ compared to that in untreated parasites. When the Ca^2+^ in the culture medium was chelated by the addition of BAPTA, we speculated that [Ca^2+^]_i_ might be reduced by PMCA function. However, the [Ca^2+^]_i_ in parasites treated with BAPTA was not reduced when compared with that in untreated parasites. These results indicate that *T. cruzi* do not constitutively import or export Ca^2+^. Therefore, Ca^2+^ released from intracellular Ca^2+^ store(s) into the cytosol by TcIP_3_R should be effectively returned to the store(s) by SERCA 24, 25.

In animal cells, [Ca^2+^]_i_ are kept at low concentrations (~ 100 nm) in the absence of extracellular stimuli 26. Phosphoinositide phospholipase C (PI‐PLC) is activated in response to signals from cell surface receptors, and it catalyzes the hydrolysis of phosphatidylinositol 4,5‐bisphosphate (PIP_2_) to generate IP_3_, which activates IP_3_R and transiently increases [Ca^2+^]_i_ 27. Our present data indicate that [Ca^2+^]_i_ in epimastigotes and amastigotes is constitutively high. Recently, it has been reported that *Trypanosoma brucei* PI‐PLC may be constitutively activated 28. Furthermore, the molecular properties of *T. cruzi* PI‐PLC have been reported to be similar to that of *T. brucei* PI‐PLC 29 (e.g., plasma membrane localization). Together, these findings suggest that constitutive activation of *T. cruzi* PI‐PLC might maintain high [Ca^2+^]_i_ through constitutive TcIP_3_R activation.

According to the cell boundary theorem, [Ca^2+^]_i_ is determined by the balance between Ca^2+^ influx and efflux, and Ca^2+^ release via IP_3_R does not result in higher [Ca^2+^]_i_ 30, 31. In mammalian cells, Ca^2+^ influx increases through the SOC mechanism activated by Ca^2+^ release from the ER, thereby resulting in an increase of [Ca^2+^]_i_ 25. Furthermore, it might be possible that [Ca^2+^]_i_ in *T. cruzi* is not always increased through TcIP_3_R directly but the parasites have some unknown mechanism(s) to increase Ca^2+^ influx. Interestingly, since amastigotes parasitize the host cell cytoplasm, where the concentration of Ca^2+^ is much lower than the [Ca^2+^]_i_ in amastigotes, the parasites may not receive Ca^2+^ from outside through a SOC‐like mechanism. However, how amastigotes maintain high [Ca^2+^]_i_ within the host cells remains unknown at present.

In conclusion, our present study revealed that basal [Ca^2+^]_i_ levels in *T. cruzi* are determined by the level of TcIP_3_R expression. Since Ca^2+^ signaling is essential for the parasite and the primary structure of TcIP_3_R shares low similarity with that of mammalian IP_3_Rs, TcIP_3_R, the key Ca^2+^ signaling molecule, is a promising drug target for Chagas disease.

## Author contributions

MH, MD, NK, KM, and NT designed the study. MH, MD, NK, KF, and HM did the experiments. MH, MD, NK, and TN wrote the manuscript. MH, MD, NK, KF, HM, YO, TS, TM, KM, and TN interpreted the data. All authors reviewed the manuscript.

## Supporting information


**Movie S1.** Changes in *T. cruzi* [Ca^2+^]_i_ within host cells. 3T3‐Swiss albino cells were infected with R‐GECO1‐expressing trypomastigotes. A bright‐field movie of cells that are heavily infected with R‐GECO1‐expressing amastigotes and trypomastigotes is shown (A). A representative microscopic movie was obtained with an inverted microscope (IX72; Olympus). Note that the trypomastigotes in the host cell move intensely. A fluorescent image of the same field is also shown (B).

## References

[1] Petersen OH , Michalak M and Verkhratsky A (2005) Calcium signalling: past, present and future. Cell Calcium 38, 161–169. 16076488 10.1016/j.ceca.2005.06.023

[2] Bootman MD , Lipp P and Berridge MJ (2001) The organisation and functions of local Ca(2+) signals. J Cell Sci 114, 2213–2222. 11493661 10.1242/jcs.114.12.2213

[3] Weber JT (2012) Altered calcium signaling following traumatic brain injury. Front Pharmacol 3, 60. 22518104 10.3389/fphar.2012.00060 PMC3324969

[4] Chatelain E (2014) Chagas disease drug discovery: toward a new era. J Biomol Screen 20, 22–35. 25245987 10.1177/1087057114550585

[5] Brener Z (1973) Biology of *Trypanosoma cruzi* . Annu Rev Microbiol 27, 347–382. 4201691 10.1146/annurev.mi.27.100173.002023

[6] Docampo R , Moreno SN and Plattner H (2014) Intracellular calcium channels in protozoa. Eur J Pharmacol 739, 4–18. 24291099 10.1016/j.ejphar.2013.11.015 PMC4037393

[7] Oberholzer M , Langousis G , Nguyen HT , Saada EA , Shimogawa MM , Jonsson ZO , Nguyen SM , Wohlschlegel JA and Hill KL (2011) Independent analysis of the flagellum surface and matrix proteomes provides insight into flagellum signaling in mammalian‐infectious *Trypanosoma brucei* . Mol Cell Proteomics 10, M111.010538. 10.1074/mcp.M111.010538 PMC3205874 21685506

[8] Luo S , Rohloff P , Cox J , Uyemura SA and Docampo R (2004) *Trypanosoma brucei* plasma membrane‐type Ca(2+)‐ATPase 1 (*TbPMC1*) and 2 (*TbPMC2*) genes encode functional Ca(2+)‐ATPases localized to the acidocalcisomes and plasma membrane, and essential for Ca(2+) homeostasis and growth. J Biol Chem 279, 14427–14439. 14724285 10.1074/jbc.M309978200

[9] Docampo R , Moreno SN and Vercesi AE (1993) Effect of thapsigargin on calcium homeostasis in *Trypanosoma cruzi* trypomastigotes and epimastigotes. Mol Biochem Parasitol 59, 305–313. 8341327 10.1016/0166-6851(93)90228-p

[10] Hashimoto M , Enomoto M , Morales J , Kurebayashi N , Sakurai T , Hashimoto T , Nara T and Mikoshiba K (2013) Inositol 1,4,5‐trisphosphate receptor regulates replication, differentiation, infectivity and virulence of the parasitic protist *Trypanosoma cruzi* . Mol Microbiol 87, 1133–1150. 23320762 10.1111/mmi.12155

[11] Hashimoto M , Nara T , Hirawake H , Morales J , Enomoto M and Mikoshiba K (2014) Antisense oligonucleotides targeting parasite inositol 1,4,5‐trisphosphate receptor inhibits mammalian host cell invasion by *Trypanosoma cruzi* . Sci Rep 4, 4231. 24577136 10.1038/srep04231 PMC3937783

[12] Hashimoto M , Morales J , Uemura H , Mikoshiba K and Nara T (2015) A novel method for inducing amastigote‐to‐trypomastigote transformation *in vitro* in *Trypanosoma cruzi* reveals the importance of inositol 1,4,5‐trisphosphate receptor. PLoS One 10, e0135726. 26267656 10.1371/journal.pone.0135726 PMC4534300

[13] Moreno SN , Silva J , Vercesi AE and Docampo R (1994) Cytosolic‐free calcium elevation in *Trypanosoma cruzi* is required for cell invasion. J Exp Med 180, 1535–1540. 7931085 10.1084/jem.180.4.1535 PMC2191711

[14] Yakubu MA , Majumder S and Kierszenbaum F (1994) Changes in *Trypanosoma cruzi* infectivity by treatments that affect calcium ion levels. Mol Biochem Parasitol 66, 119–125. 7984174 10.1016/0166-6851(94)90042-6

[15] Lammel EM , Barbieri MA , Wilkowsky SE , Bertini F and Isola EL (1996) *Trypanosoma cruzi*: involvement of intracellular calcium in multiplication and differentiation. Exp Parasitol 83, 240–249. 8682192 10.1006/expr.1996.0070

[16] Zhao Y , Araki S , Wu J , Teramoto T , Chang YF , Nakano M , Abdelfattah AS , Fujiwara M , Ishihara T , Nagai T *et al* (2011) An expanded palette of genetically encoded Ca^2+^ indicators. Science 333, 1888–1891. 21903779 10.1126/science.1208592 PMC3560286

[17] Borges‐Pereira L , Budu A , McKnight CA , Moore CA , Vella SA , Hortua Triana MA , Liu J , Garcia CR , Pace DA and Moreno SN (2015) Calcium signaling throughout the *Toxoplasma gondii* lytic cycle: a study using genetically encoded calcium indicators. J Biol Chem 290, 26914–26926. 26374900 10.1074/jbc.M115.652511 PMC4646405

[18] Borges‐Pereira L , Campos BR and Garcia CR (2014) The GCaMP3 – A GFP‐based calcium sensor for imaging calcium dynamics in the human malaria parasite *Plasmodium falciparum* . MethodsX 1, 151–154. 26150947 10.1016/j.mex.2014.08.005 PMC4472923

[19] Vazquez MP and Levin MJ (1999) Functional analysis of the intergenic regions of TcP2beta gene *loci* allowed the construction of an improved *Trypanosoma cruzi* expression vector. Gene 239, 217–225. 10548722 10.1016/s0378-1119(99)00386-8

[20] Lorenzi HA , Vazquez MP and Levin MJ (2003) Integration of expression vectors into the ribosomal locus of *Trypanosoma cruzi* . Gene 310, 91–99. 12801636 10.1016/s0378-1119(03)00502-x

[21] Iizumi K , Mikami Y , Hashimoto M , Nara T , Hara Y and Aoki T (2006) Molecular cloning and characterization of ouabain‐insensitive Na(+)‐ATPase in the parasitic protist, *Trypanosoma cruzi* . Biochim Biophys Acta 1758, 738–746. 16797482 10.1016/j.bbamem.2006.04.025

[22] Nakajima‐Shimada J , Hirota Y and Aoki T (1996) Inhibition of *Trypanosoma cruzi* growth in mammalian cells by purine and pyrimidine analogs. Antimicrob Agents Chemother 40, 2455–2458. 8913446 10.1128/aac.40.11.2455 PMC163557

[23] Gluenz E , Taylor MC and Kelly JM (2007) The *Trypanosoma cruzi* metacyclic‐specific protein Met‐III associates with the nucleolus and contains independent amino and carboxyl terminal targeting elements. Int J Parasitol 37, 617–625. 17239886 10.1016/j.ijpara.2006.11.016 PMC2424140

[24] Parekh AB and Putney JW Jr (2005) Store‐operated calcium channels. Physiol Rev 85, 757–810. 15788710 10.1152/physrev.00057.2003

[25] Smyth JT , Dehaven WI , Jones BF , Mercer JC , Trebak M , Vazquez G and Putney JW Jr (2006) Emerging perspectives in store‐operated Ca^2+^ entry: roles of Orai, Stim and TRP. Biochim Biophys Acta 1763, 1147–1160. 17034882 10.1016/j.bbamcr.2006.08.050

[26] Syntichaki P and Tavernarakis N (2003) The biochemistry of neuronal necrosis: rogue biology? Nat Rev Neurosci 4, 672–684. 12894242 10.1038/nrn1174

[27] Berridge MJ (1993) Inositol trisphosphate and calcium signalling. Nature 361, 315–325. 8381210 10.1038/361315a0

[28] King‐Keller S , Moore CA , Docampo R and Moreno SN (2015) Ca^2+^ regulation of *Trypanosoma brucei* phosphoinositide phospholipase C. Eukaryot Cell 14, 486–494. 25769297 10.1128/EC.00019-15 PMC4421009

[29] de Paulo Martins V , Okura M , Maric D , Engman DM , Vieira M , Docampo R and Moreno SN (2010) Acylation‐dependent export of *Trypanosoma cruzi* phosphoinositide‐specific phospholipase C to the outer surface of amastigotes. J Biol Chem 285, 30906–30917. 20647312 10.1074/jbc.M110.142190 PMC2945582

[30] Ríos E (2010) The cell boundary theorem: a simple law of the control of cytosolic calcium concentration. J Physiol Sci 60, 81–84. 19937486 10.1007/s12576-009-0069-z PMC2821834

[31] Friel DD and Tsien RW (1992) A caffeine‐ and ryanodine‐sensitive Ca^2+^ store in bullfrog sympathetic neurones modulates effects of Ca^2+^ entry on [Ca^2+^]_i_ . J Physiol 450, 217–246. 1432708 10.1113/jphysiol.1992.sp019125 PMC1176120

